# Stromal Derived Factor-1 (SDF-1/CXCL12) and CXCR4 in renal cell carcinoma metastasis

**DOI:** 10.1186/1476-4598-5-56

**Published:** 2006-11-03

**Authors:** Judong Pan, Javier Mestas, Marie D Burdick, Roderick J Phillips, George V Thomas, Karen Reckamp, John A Belperio, Robert M Strieter

**Affiliations:** 1 Department of Medicine, David Geffen School of Medicine at UCLA, 900 Veteran Ave., Los Angeles, California, USA; 2 Department of Pathology, David Geffen School of Medicine at UCLA, 900 Veteran Ave., Los Angeles, California, USA; 3 Department of Pediatrics, David Geffen School of Medicine at UCLA, 900 Veteran Ave., Los Angeles, California, USA

## Abstract

Renal cell carcinoma (RCC) is characterized by organ-specific metastases. The chemokine stromal derived factor-1 (SDF-1/CXCL12) and its receptor CXCR4 have been suggested to regulate organ-specific metastasis in various other cancers. On this basis, we hypothesized that the biological axis of CXCL12 via interaction with its receptor, CXCR4, is a major mechanism for RCC metastasis. We demonstrated that CXCR4 was significantly expressed on circulating cytokeratin+ RCC cells from patients with known metastatic RCC. We detected up-regulation of CXCR4 mRNA and protein levels on a human RCC cell line by either knockdown of the von Hippel-Lindau (VHL) tumor suppressor protein, or incubating the cells under hypoxic conditions. The enhanced CXCR4 expression was mediated through the interaction of the Hypoxia Inducible Factor-1α (HIF-1α) with the promoter region of the CXCR4 gene. Furthermore, the expression of CXCR4 on human RCC directly correlated with their metastatic ability *in vivo *in both heterotopic and orthotopic SCID mouse models of human RCC. Neutralization of CXCL12 in SCID mice abrogated metastasis of RCC to target organs expressing high levels of CXCL12; without altering tumor cell proliferation, apoptosis, or tumor-associated angiogenesis. Therefore, our data suggest that the CXCL12/CXCR4 biological axis plays an important role in regulating the organ-specific metastasis of RCC.

## Background

Renal cell carcinoma (RCC) accounts for approximately 3% of new cancer incidence and mortality in the U.S. [1]. In general, approximately a third of the patients at time of presentation have metastatic RCC (mRCC), and another third that present with local disease will eventually experience recurrence and metastases with a with a median survival of less than one year [2]. The mortality and morbidity of RCC is strongly associated with its high propensity to metastasize to specific organs. To explain the specific pattern of tumor metastases, it has been demonstrated that sites of metastases are determined not only by the characteristics of neoplastic cells but also by the microenvironment of the specific organ [3]. In a similar manner to leukocyte trafficking, the target organs for metastatic events express constitutive levels of chemoattractants that mediate extravasation of tumor cells. Recently, extensive studies have suggested that chemokines may play a major role in mediating tumor metastasis [4-8].

Chemokines are a superfamily of small (8–10 kD) proteins, which play a pivotal role in the regulation of leukocyte trafficking and extravasation into sites of tissue inflammation [9-13]. Different cancers are found to express several chemokine receptors, and their corresponding ligands are expressed at sites of tumor metastases [6,7,14,15]. However, CXCR4 appears to be the major chemokine receptor expressed on cancer cells [4,5,8]. CXCR4 was originally discovered as the co-receptor for lymphotropic strains of HIV [16] and CXCL12 (stromal derived factor-1, SDF-1) is its lone ligand [17]. CXCL12 has been found to be secreted by bone marrow stromal cells and is important during embryogenesis for the colonization of bone marrow by HSC [18]. It is also essential in adult life for retention/homing of HSC [19]. Both CXCL12^-/- ^and CXCR4^-/- ^mice die *in utero *with defects in heart, brain and large vessel development [20-24]. The role of CXCL12/CXCR4 axis in organ-specific metastasis was initially suggested in breast cancer [6]. Since then, CXCR4 expression has been reported in at least twenty three epithelial, mesenchymal and hematopoietic cancers, suggesting the importance of this ligand/receptor axis, in general in tumor metastasis [4,5,8]. In addition, studies have also suggested that CXCL12/CXCR4 may indirectly promote tumor metastases by mediating proliferation of tumor cells and enhancing tumor-associated angiogenesis [25-32].

While increasing evidence has suggested the pivotal role of CXCL12/CXCR4 biological axis in tumor metastasis, the specific mechanisms regulating CXCR4 expression in different tumors are poorly understood. Recently, Hypoxia Inducible Factor-1α (HIF-1α) has been found to be a critical transcription factor for gene expression of CXCR4 in RCC [33,34]. Moreover, von Hippel-Lindau tumor suppressor gene (VHL), the most common mutated gene in RCC, was found to negatively regulate the expression of CXCR4, owing to its capacity to target HIF-1α for degradation under normoxic conditions [33,34]. More recently, we showed that both EGF and hypoxia can induce CXCR4 expression in non-small cell lung cancer (NSCLC) cells via the VHL/HIF-1α axis and this process is regulated by both the PI3-kinase/PTEN/AKT/mTor pathway and hypoxia [35]. These findings led to the hypothesis that CXCR4 is a biomarker that predicts the metastatic potential of RCC, and that the CXCL12/CXCR4 biological axis is regulated by VHL/HIF-1α in RCC and is a major mechanism for trafficking of RCC to metastatic sites.

In an effort to address the potential role of CXCR4 as a biomarker for predicting the metastatic potential of RCC, we first measured CXCR4 expression on circulating cytokeratin+ cells in patients with mRCC, and found significantly increased levels of cytokeratin+ cells that co-expressed CXCR4, as compared to normal human subjects. CXCR4 mRNA and protein levels were markedly up-regulated in human RCC cell lines, in which VHL was stably knocked down via RNA interference. The expression of CXCR4 in these cells could be further augmented in the presence of hypoxia, and was functional in terms of chemotaxis in response to CXCL12. Our results further demonstrated that the enhanced CXCR4 expression induced by both conditions was mediated through the binding of HIF-1α to the CXCR4 promoter region, which lead to increased transcription of the CXCR4 gene. The expression of CXCR4 on human RCC correlated with their metastatic ability in both heterotopic and orthotopic SCID mouse models, and treatment with specific anti-CXCL12 antibodies markedly abrogated metastasis of RCC to target organs, expressing high levels of CXCL12, in the orthotopic model of RCC without significant changes in tumor cell proliferation, tumor cell apoptosis, or tumor-associated angiogenesis of the primary tumor. The findings of this study support the notion that the CXCL12/CXCR4 biological axis plays a critical role in regulating organ-specific metastasis of RCC.

## Results

### CXCR4 is significantly expressed on circulating cytokeratin positive cells in patients with mRCC

**Figure 1:**
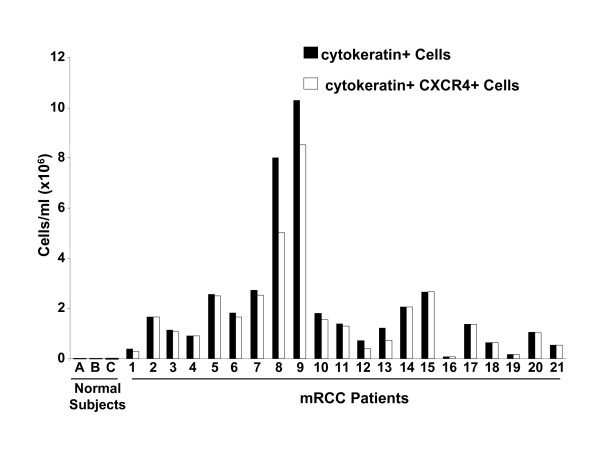
Expression of CXCR4 on circulating pan-cytokeratin positive cells in patients with mRCC as determined by FACS analysis. Cells isolated from the buffy coat of mRCC patients were stained with pan-cytokeratin (dark-colored boxes) or stained with both pan-cytokeratin and CXCR4 for dual color analysis (light-colored boxes). Specimens from three normal human subjects were included as controls.

Recent studies have shown that the CXCL12/CXCR4 biological axis is important in trafficking malignant cells in an organ-specific manner [4,5,8], and that hypoxia through HIF-1α/VHL appears to modulate the expression of CXCR4 on malignant cells [33-35]. However, CXCR4 has not been previously shown to be expressed on metastatic circulating mRCC cells. We therefore hypothesized that CXCR4 would be expressed on a circulating population of cells compatible with mRCC. We enrolled 21 patients with mRCC into a pilot study, as well as three normal volunteer control subjects, and obtained 10 mls of heparinized blood and isolated the buffy-coat cells for FACS analysis of cytokeratin and dual color analysis of CXCR4 expression. As shown in Fig. 1, we found a marked increase in cytokeratin positive cells in the circulation of patients with mRCC, as compared to normal control subjects. A large variation in the number of circulating cytokeratin+ cells and cytokeratin+ CXCR4+ cells could be observed in mRCC patients, with more than 50-fold difference between the patient with smallest number of stained cells and the patient with largest number of these cells. When the cytokeratin positive cells from patients with mRCC were examined for expression of CXCR4, > 90% were found to be CXCR4 positive. In the three normal control subjects, the levels of cytokeratin positive cells, although detectable, were extremely low and compatible with the presence of cytokeratin+ cells in normal subjects [36,37]. These findings support the notion that the majority of potential circulating RCC cells express CXCR4, and may serve as a foundation for further studies to define whether the presence and magnitude of circulating mRCC cells are prognostic in patients with RCC.

### Hypoxia plays a significant role in the regulation of CXCR4 on human RCC

**Figure 2:**
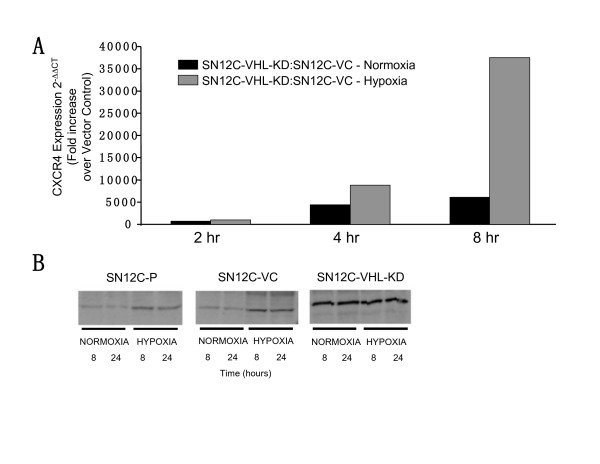
Hypoxia and VHL knockdown enhance CXCR4 expression. (A). SN12C-VHL-KD cells were serum-starved and exposed to either normoxia or hypoxia (94% N_2_, 5% CO_2 _and 1% O_2_) for the times indicated. Changes in gene expression were then determined by real-time quantitative PCR. Fold induction represents increases in CXCR4 expression in SN12C-VHL-KD cells compared to SN12C cells transfected with vector control (SN12C-VC). (B). SN12C-P, SN12C-VC and SN12C-VHL-KD cells were treated with either normoxia or hypoxia as described in (A) for the times indicated and then subjected to Western analysis to examine changes in CXCR4 protein levels.

Recently, the expression of CXCR4 on RCC and NSCLC tumor cells was shown to be regulated by hypoxia and the VHL/HIF-1α pathway [33-35]. To further characterize the role of hypoxia and/or VHL/HIF-1α in the regulation of the expression of CXCR4 on RCC cells and to determine whether this chemokine receptor is a critical factor for promoting RCC metastases, we next exposed SN12C-P, SN12C-VC and SN12C-VHL-KD cells to normoxia or hypoxia for 2, 4, 8, and 24 hours and isolated mRNA and protein for quantitative TaqMan RT-PCR and Western blot analysis of CXCR4, respectively. As shown in Fig 2A, SN12C-VHL-KD cells demonstrated a marked increase in CXCR4 gene expression under both normoxic and hypoxic conditions, as compared to SN12C-VC. Similar results were found when comparing the SN12C-VHL-KD to SN12C-P (data not shown). Total cellular levels of CXCR4 protein paralleled the expression of mRNA in SN12C-VHL-KD cells with significantly higher levels of CXCR4 protein found under both conditions of normoxia and hypoxia, as compared to either SN12C-P or SN12C-VC cell lines (Fig. 2B). These latter two cell lines demonstrated markedly increased CXCR4 protein levels only under hypoxic conditions (Fig. 2B). CXCR4 cell surface expression was seen by FACS analysis and correlated with the results obtained from Western blot analysis (data not shown).

### VHL knockdown and hypoxia increase the chemotactic responsiveness of RCC cells to CXCL12

**Figure 3:**
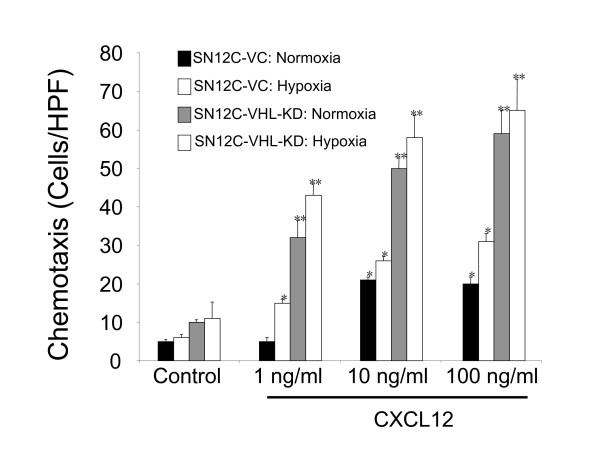
SN12C-VC and SN12C-VHL-KD chemotaxis in the presence of CXCL12 (1, 10 and 100 ng/ml) after 24 hours incubation under normoxic or hypoxic conditions as described previously. Results are expressed as the number of cells that migrated per high-power field (HPF). Data represent the mean ± SEM from five HPFs.

After confirming the role of the HIF-1α/VHL axis in the up-regulation of CXCR4 mRNA and protein expression, we assessed whether this led to functional differences in migration in response to the CXCR4 ligand, CXCL12. SN12C-P, SN12C-VC, and SN12C-VHL-KD cells were exposed to either normoxia or preconditioned with hypoxia for 24 hours followed by analysis of chemotaxis in response to CXCL12. SN12C-VHL-KD cells demonstrated a dose-dependent increase in chemotaxis in response to CXCL12 under both conditions of normoxia and hypoxia (Fig. 3). Hypoxia preconditioning of the SN12C-VC and SN12C-P cells also resulted in a dose-dependent increase in chemotactic activity (Fig. 3 and data not shown). The levels of chemotactic activities of SN12C-VC were not significantly different from those of SN12C-P cells under either normoxic or hypoxic conditions (data not shown). These results confirmed that the loss of function of VHL results in augmented chemotactic activity in response to CXCL12. Moreover, in cells with functional VHL, hypoxia plays a role to increase this response.

### VHL knockdown and hypoxia activate HIF-1α expression in human RCC cells and promote HIF-1-mediated transcription of the CXCR4 promoter

**Figure 4:**
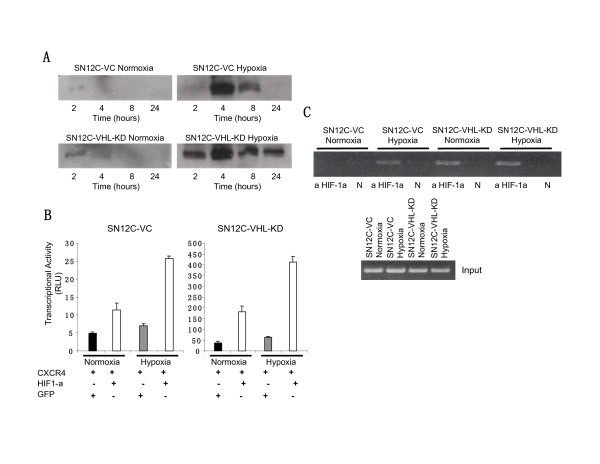
VHL knockdown and the condition of hypoxia induce activation of HIF-1 transcription and transactivation of the CXCR4 promoter. (A). SN12C-VC and SN12C-VHL-KD cells were serum-starved and exposed to either normoxia or hypoxia for the times indicated. Subsequently, nuclear extracts were prepared and analyzed by SDS-PAGE and Western blotting with a specific mouse-anti-human HIF-1α monoclonal antibody. (B). SN12C-VC and SN12C-VHL-KD cells were transfected with a reporter construct comprising a 2.6-kilobase fragment of the CXCR4 promoter and a separate Renilla control reporter. Cells were co-transfected with either a control vector expressing GFP or a vector expressing HIF-1α. The transfected cells were subsequently exposed to either normoxia or hypoxia for 8 hours. Cell extracts were prepared and analyzed in a luminometer. Relative light units (RLU) were normalized to the Renilla control. (C). SN12C-VC and SN12C-VHL-KD cells were serum-starved and exposed to either normoxia or hypoxia for 4 h. ChIP assay was subsequently performed to investigate the recruitment of HIF-1α on the CXCR4 promoter. Precleared chromatin solutions were immunoprecipitated with either mouse anti-human HIF-1α monoclonal antibody or with no antibody as negative controls (N). The lower panel represents input genomic DNA before addition of antibody.

Having established that VHL knockdown and/or hypoxia are capable of up-regulating CXCR4 expression in SN12C cells, we next wanted to examine the underlying mechanism that mediates this effect. It has been reported that the VHL tumor suppressor protein negatively regulates CXCR4 expression owing to its capacity to target HIF-1α for degradation under normoxic conditions, and that CXCR4 induction by hypoxia is dependent on the transactivation of HIF-1α [33-35]. Thus, we exposed SN12C-VC and SN12C-VHL-KD cells to either normoxia or hypoxia for the times indicated and then examined intranuclear HIF-1α expression by Western analysis (Fig. 4A). Under normoxic conditions little or no intranuclear expression of HIF-1α was observed in SN12C-VC cells, whereas the expression of HIF-1α was detectable in SN12C-VHL-KD cells within 2 hours. Under hypoxic conditions, strong intranuclear expression of HIF-1α was observed in both SN12C-VC and SN12C-VHL-KD cells. The expression of HIF-1α in SN12C-VHL-KD cells, however, could be observed as early as 2 hours after exposure to hypoxia when HIF-1α was barely detectable in SN12C-VC cells; and remained elevated at 8 to 24 hours after exposure to hypoxia, when HIF-1α elevation was no longer detected in SN12C-VC cells (Fig. 4A).

To further verify that HIF-1α contributed to the transcription and up-regulation of CXCR4 gene expression, we transfected the SN12C-VC and SN12C-VHL-KD cells with a luciferase reporter construct containing a 2.6-kb fragment of the CXCR4 promoter as well as either a random control GFP cDNA or HIF-1α cDNA as previously described [35] (Fig. 4B). Transfected cells were then exposed to either hypoxic or normoxic conditions. Under these conditions significant transactivation of the CXCR4 promoter was observed in those cells receiving the HIF-1α cDNA but not the GFP cDNA, and those cells exposed to hypoxia but not normoxia (Fig. 4B). Furthermore, significant activation of CXCR4 promoter could be detected in SN12C-VHL-KD cells even under normoxic conditions and in the absence of HIF-1α cDNA transfection. This activation was further enhanced by either hypoxic preconditioning or transfection with HIF-1α cDNA (Fig. 4B).

Next, to obtain direct evidence for the physical interaction between HIF-1α and the CXCR4 promoter, we performed a chromatin immunoprecipitation assay to determine whether VHL knockdown and/or hypoxia promoted the binding of the HIF-1α transcription factor to its cognate DNA binding motif on the CXCR4 promoter region (Fig. 4C). We exposed SN12C-VC and SN12C-VHL-KD cells to normoxia or hypoxia for 4 hours and then prepared, precleared and sonicated chromatin solutions for analysis. Our results indicate that under normoxic conditions there is little or no inducible binding of HIF-1α to the CXCR4 promoter in SN12C-VC cells, whereas a 4-hour exposure to hypoxia mediated a much stronger interaction between HIF-1α and the CXCR4 promoter in these cells (Fig. 4C). In contrast, significant recruitment of HIF-1α to the CXCR4 promoter could be observed in SN12C-VHL-KD cells under both normoxia and hypoxia (Fig. 4C), suggesting the strong transactivation of CXCR4 promoter by the HIF-1α transcription factor in the absence of regulation by the tumor suppressor protein VHL.

### The expression of CXCR4 on human RCC correlates with their metastatic ability in both heterotopic and orthotopic models of human RCC metastasis in SCID mice

**Figure 5:**
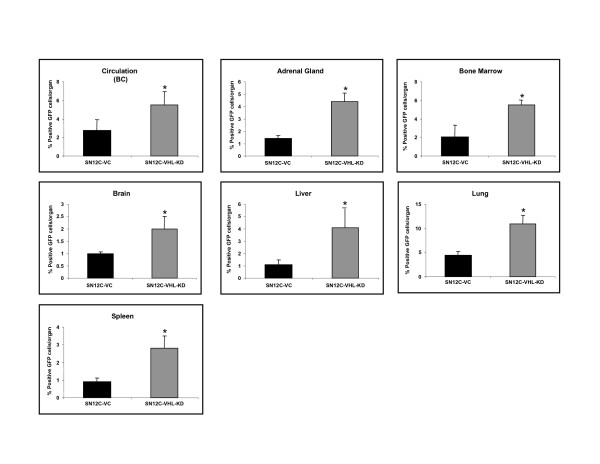
The expression of CXCR4 on human RCC cells correlates with their ability to metastasize to specific organs in SCID mice bearing orthotopic (A) or heterotopic (B) tumors. SCID mice were subjected to orthotopic or heterotopic xenoengraftment of either SN12C-VC or SN12C-VHL-KD cells. Six animals were included in each group. Data are expressed as the percentage of GFP+ cells found in each organ examined by FACS analysis. Data represent mean ± SEM.

Since our above studies demonstrated that hypoxia and the HIF-1α/VHL axis was critical in the regulation of CXCR4 expression and function in RCC cell lines, and our previous study identified the major organs that express elevated levels of CXCL12 (i.e., lungs, adrenal glands, bone marrow, liver, and brain, as compared to the primary tumors) [7]; we next wanted to determine whether alteration of VHL expression by knockdown could lead to changes in metastatic behavior of RCC. Since SN12C-VC cells demonstrated the same properties as the parental SN12C-P cells in terms of the CXCL12/CXCR4 biological axis in our *in vitro *studies (Fig. 2, Fig. 3 and data not shown), we performed the following *in vivo *studies using only SN12C-VC and SN12C-VHL-KD cells. We developed both an orthotopic RCC tumor model using 10^4 ^SN12C-VHL-KD or SN12C-VC cells expressing GFP directly injected into the subcapsular region of the left kidney and a heterotopic model using 10^6 ^SN12C-VHL-KD or SN12C-VC cells expressing GFP injected into the flank of SCID mice. Mice bearing tumors were sacrificed after 4 weeks, and their major organs were harvested and processed to assess single cell suspensions for the expression of GFP by FACS analysis. GFP labeling of the RCC cells allowed us to quantitatively assess the magnitude of metastatic lesions in various organs. As shown in Fig. 5A, we found that SN12C-VHL-KD, as compared to SN12C-VC orthotopic tumors had a greater propensity to metastasize to specific sites, and were found to have higher numbers of cells in circulation/buffy coat, adrenal glands, bone marrow, brain, liver, lung, kidney and spleen. The number of GFP positive cells in the primary tumors, however, did not differ significantly between these two groups (data not shown). Consistent with our findings in the orthotopic model, we found similar results for the same human RCC cells lines inoculated in the heterotopic position in SCID mice (Fig. 5B).

### Depletion of CXCL12 inhibits RCC metastasis

**Figure 6:**
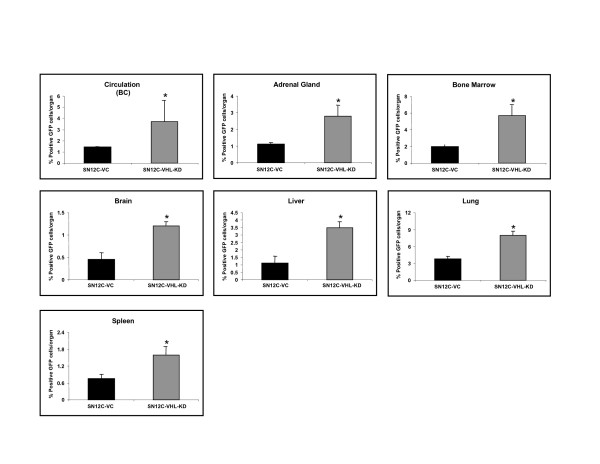
Neutralization of CXCL12 in animals bearing orthotopic human RCC tumors resulted in attenuated metastasis to specific organs. SCID mice bearing orthotopic xenoengraftment of either SN12C-VC or SN12C-VHL-KD cells were subjected to intraperitoneal injection of anti-CXCL12 antibodies or preimmune serum as a control. Six animals were included in each treatment group. Data are expressed as the percentage of GFP+ cells found in each organ examined by FACS analysis. Data represent mean ± SEM.

Since the expression of CXCR4 correlated to the magnitude of metastatic RCC cells in specific organs, we next wanted to determine whether depletion of CXCL12 by specific neutralizing antibodies to CXCL12 in SCID mice bearing SN12C-VHL-KD and SN12C-VC orthotopic tumors expressing GFP would attenuate RCC metastasis. Having established the specificity of the anti-CXCL12 antibody [7,38], we next injected SN12C-VC or SN12C-VHL-KD cells directly into the subcapsular region of the left kidney of SCID mice, then treated the mice with intraperitoneal injections (500 μl) of either neutralizing goat anti-CXCL12 or preimmune serum, three times per week for 4 weeks, starting at the time of tumor cell inoculation. At time of sacrifice, cells from different organs were isolated and examined for spontaneous metastases as assessed by FACS analysis of GFP+ tumor cells. We found that animals bearing the human tumor cell lines treated with neutralizing anti-CXCL12 antibodies resulted in markedly reduced metastases to the lungs, adrenal glands, liver and circulation (buffy coat), as compared to control antibody treated groups (Fig. 6). Furthermore, in the context of the VHL knockdown cell line, the magnitude of metastasis of this cell line was reduced to a similar level as the vector control cells in the presence of anti-CXCL12 (Fig. 6).

### Depletion of CXCL12 was not associated with a change in primary tumor vascular density, tumor cell proliferation or apoptosis

Having established that CXCL12 depletion significantly impaired the metastasis of RCC to specific target organs, we next wanted to determine whether this effect was mediated by CXCL12/CXCR4 biological axis on RCC metastases, or by alterations of RCC tumor or tumor cell biology, such as tumor-associated vascular density, or tumor cell proliferation and/or apoptosis. Orthotopic tumor sections of mice bearing SN12C-VC and SN12C-VHL-KD tumors were stained using anti-Factor VIII-related antigen antibody, anti-PCNA antibody, or TUNEL method for the morphometric evaluation of tumor-associated vascular density, proliferation, or apoptosis, respectively. As shown in Table 1, the vascular density in primary SN12C-VC or SN12C-VHL-KD tumors from SCID mice as detected by Factor-VIII-related antigen staining was not significantly different in animals treated with anti-CXCL12 antibodies, as compared to animals treated with control antibodies. The number of SN12C-VC or SN12C-VHL-KD tumor cells undergoing proliferation or apoptosis, as determined by PCNA and TUNEL staining, respectively, was not significantly different between the anti-CXCL12 and the control antibody treated mice (Table 1). In addition, we examined the proliferation and apoptosis of SN12C-VC and SN12C-VHL-KD cells *in vitro *in the presence of various concentrations of CXCL12 or neutralizing anti-CXCR4 antibody by [^3^H]thymidine incorporation assay and TUNEL assay, respectively. We found that the proliferative or apoptotic properties of SN12C-VC and SN12C-VHL-KD cells were not altered by either stimulation or inhibition of CXCL12/CXCR4 biological axis (data not shown).

**Table 1:** RCC tumor-associated angiogenesis (Factor VIII-related antigen), proliferation (PCNA), and apoptosis (TUNEL) in SCID mice treated with anti-CXCL12

Group	FVIIIRA^A^+/hpf	PCNA+/hpf	TUNEL+/hpf
VC CTRL	9.3 ± 1.3	40.4 ± 6.6	9.2 ± 1.1
VC αCXCL12	6.6 ± 0.8	44.7 ± 3.1	6.7 ± 1.0
	*p *= 0.19	*p *= 0.60	*p *= 0.15
KD CTRL	26.7 ± 1.4	107.5 ± 4.8	28.9 ± 2.5
KD αCXCL12	30.1 ± 1.8	116.6 ± 7.6	23.4 ± 3.0
	*p *= 0.13	*p *= 0.29	*p *= 0.17

Factor VIII-related antigen stained vessels or PCNA or TUNEL stained cells were counted in 5–10 high power fields (hpf; ×400) in three sections from each group. ^A ^FVIIIRA: Factor VIII-related antigen. CTRL = control serum. αCXCL12 = specific neutralizing anti-CXCL12 serum.

## Discussion

RCC ranks as the 10^th ^leading cause of cancer death [39]. The morbidity and mortality of this disease is mainly due to its significant propensity to metastasize in an organ-specific manner. This metastatic pattern is also shared by various other malignancies, such as breast cancer and NSCLC [6,7]. Müller and colleagues provided initial evidence linking the CXCL12/CXCR4 biological axis to breast cancer metastasis to specific organs [6], which was confirmed in non-small cell lung cancer [7]. More recent studies have suggested that CXCR4 is expressed on various other cancer cells and its expression stimulates migration of cancer cells towards a CXCL12 gradient established in specific target organs [4,5,8]. Furthermore, elevated CXCR4 expression was detected in several human RCC cell lines and tumor samples, while only minimal CXCR4 expression was detected in normal kidney tissues [40]. While these findings suggested that the CXCL12/CXCR4 biological axis may be a critical determinant for the metastatic potential of RCC, studies demonstrating a direct link of CXCR4 expression and metastatic potential remained to be elucidated in response to organ-specific expression of CXCL12.

Given that CXCR4 mediates the trafficking of breast and non-small cell lung cancer cells in an organ-specific manner [4,5,8], we hypothesized that there would be an increase in the number of RCC cells in circulation in patients with mRCC, and that these cells would express CXCR4. Upon analysis of buffy coats from patients with known mRCC, we indeed found that there was a marked increase in the number of cytokeratin+ cells in circulation compared to normal subjects, suggesting that these cells were comparable with circulating malignant cells. The number of circulating cytokeratin+ cells varied greatly among patients, however in most cases circulating cytokeratin+ cells made up a significant portion of total blood cell counts (data not shown), indicative of disease progression [41,42]. The low levels of cytokeratin+ cells in normal subjects may be due to expression of cytokeratin by bone marrow-derived stem cell subpopulations, which has previously been described [36,37,43]. When the cytokeratin+ cells from patients with mRCC were examined for expression of CXCR4, > 90% of them were found to be CXCR4 positive. These findings suggest that CXCR4 is a predominant biomarker on cytokeratin+ cells in the circulation of patients with mRCC and the presence of CXCR4 expression on these cells may directly correlate with mRCC. Furthermore, these studies may support future clinical trials that use the presence and magnitude of cytokeratin+ CXCR4+ cells as a prognostic biomarker in patients with RCC.

Recent studies have suggested that hypoxia, particularly HIF-1α, regulates the expression of CXCR4 in RCC [33,34,44]. These studies further suggest that the loss or functional inactivation of the protein product of VHL, a tumor suppressor gene commonly mutated in clear cell RCC, results in persistent activation of HIF-1α and a dramatic increase in CXCR4 expression due to loss of its ability to target HIF-1α for degradation by the 26S proteasome [33,34,44]. In support of these studies, we demonstrated that either knocking down VHL expression in SN12C cells (i.e. cells that normally express VHL) or exposing these cells to hypoxic conditions led to markedly increased expression of CXCR4 mRNA and protein. Furthermore, our results confirmed that the up-regulation of CXCR4 expression in RCC induced by these conditions was mediated by the direct physical interaction between the HIF-1α transcription factor and its specific binding sites on the CXCR4 promoter region, which is probably the hypoxia response element (HRE), as suggested by previous studies [33,35,45]. This is consistent with our recent findings in NSCLC, in which the combination of hypoxia and activation of the tyrosine kinase receptor, EGFR, led to additive or synergistic up-regulation of CXCR4 expression via HIF-1α [35]. EGFR activation/signaling and hypoxia, unlike the VHL mutation, are characteristic of many cancers. Therefore these findings suggest that tyrosine kinase receptor activation and/or a response to hypoxia, leading to increased HIF-1α, is critical for the regulation of the expression of CXCR4, and may represent a general scheme in tumor metastasis.

Since previous studies had found a correlation of CXCR4 expression on metastatic RCC cells, but had not shown a direct link of CXCL12 and metastasis of CXCR4-expressing RCC cells, we then examined whether VHL knockdown, and subsequently CXCR4 expression, could change the metastatic potential of these human RCC cell lines in SCID mice. Our results showed markedly increased metastases of human RCC cells in an organ-specific manner to adrenal glands, buffy coat, bone marrow, brain, kidney, spleen, liver and the lungs of mice bearing SN12C-VHL-KD tumors, as compared to those bearing SN12C-VC tumors, in both heterotopic and orthotopic tumor models, using a sensitive analysis of GFP as a marker of metastatic human cells in murine organs. Most of these organs have been found to express elevated levels of CXCL12 in SCID mice [6,7]. Moreover, targeting CXCL12 with a neutralizing antibody significantly reduced the metastatic potential of the human RCC cell lines in the orthotopic model. Therefore, our study provided initial evidence in an *in vivo *model system that directly links the VHL/HIF-1α axis to CXCL12/CXCR4-mediated organ-specific metastases of human RCC.

CXCL12 is the lone ligand for the chemokine receptor, CXCR4. Previously it was thought that CXCL12 bound only to CXCR4. Therefore, targeting of CXCL12 allowed us to investigate the role of the CXCL12/CXCR4 biological axis in RCC metastasis *in vivo*. Moreover, this strategy conferred the advantage of avoiding possible RCC cell clearance from the circulation via antibody-dependent cellular cytotoxicity, through the use of anti-CXCR4 antibodies. However, recently it has been reported that CXCL12 is also a ligand for the orphan receptor RDC1/CXCR7 in T lymphocytes [46]. Although the distribution and biology of CXCR7 remains to be elucidated, it is possible that CXCR7 plays a role in CXCL12-mediated tumor metastasis. If this is indeed the case, then our strategy of targeting the ligand for both CXCR4 and CXCR7 (CXCL12) allowed us to block both the biology of CXCR4 and CXCR7 *in vivo*, and provided a single-target strategy by which to decrease the metastatic potential of RCC. Whether our results are due to inhibiting both CXCR4 and CXCR7 or due solely to inhibition of CXCR4 remains to be determined.

Although in this study we focused on the effect of VHL inactivation on CXCL12/CXCR4-mediated organ-specific metastases, it is possible that other hypoxia-inducible genes under the control of HIF-1α/VHL pathway may also play a role in tumor metastasis. Increased HIF-1α has been shown to regulate a host of genes involved in cellular processes such as proliferation, survival, glucose metabolism, and angiogenesis [47-50]. For example, vascular endothelial growth factor (VEGF) has been shown to be negatively regulated by pVHL [51,52], and its overexpression is associated with increased tumor angiogenesis and augmented metastasis in RCC patients [53]. However, we found that SCID mice bearing orthotopic SN12C-VHL-KD tumors demonstrated significantly reduced RCC metastasis to specific organs when CXCL12 was depleted by specific neutralizing antibodies, demonstrating the importance of CXCL12 biology in mediating tumor metastasis.

While our data and other studies have suggested the important role of CXCL12/CXCR4 biological axis in organ-specific tumor metastasis, studies have also suggested that CXCL12/CXCR4 may be implicated in promoting angiogenesis, tumor cell proliferation and survival [25-32]. Interestingly, it has been demonstrated that both RCC cell lines and human RCC tumor specimens express elevated levels of CXCR4, but not its ligand CXCL12, as compared to normal kidney specimens [40]. Our findings demonstrate that tumor-associated angiogenesis, proliferation and apoptosis are not affected in the primary RCC tumors in animals treated with neutralizing anti-CXCL12 antibodies, as compared to control antibodies. This is consistent with our previous study using a NSCLC-bearing mouse model [7], and indicates that the function of CXCL12 may not be as important in promoting angiogenesis, proliferation, and cell survival in the local tumor microenvironment of RCC. However, although we see a significant reduction in the organ-specific metastasis of RCC cells in response to CXCL12 depletion, we cannot exclude the possibility that small amounts of non-neutralized CXCL12 remain in the tumor microenvironment and may contribute to angiogenic and proliferative responses within the primary tumor.

## Conclusion

In conclusion, our studies demonstrate for the first time the direct link of CXCL12 related to CXCR4-expressing RCC cells in promoting their metastatic potential. CXCR4 detected on circulating cytokeratin positive cells in patients with mRCC may represent a biomarker for the metastatic potential of these cells. Future studies will determine whether this marker can be used to identify patients with subclinical metastases. Furthermore, strategies to attenuate the CXCL12/CXCR4 biological axis may be employed to inhibit the metastatic potential of these cells *in vivo*. In fact, small molecule antagonists to CXCR4, such as AMD3100, and RNA interference strategies have already been developed to inhibit the binding of lymphotropic strains of HIV to CXCR4 on T cells [54-57]. Additionally, HIF1α antagonists are also in development and clinical studies are planned in clear cell RCC [58]. Therefore, in the future, we could see pharmaceutical reagents used to block the CXCL12/CXCR4 biological axis and impact on RCC metastasis.

## Methods

### Reagents

Polyclonal goat anti-CXCL12 antibodies were produced by immunization with recombinant CXCL12 (PeproTech, Rocky Hill, NJ) as previously described [7,59]. Five hundred microliters of anti-CXCL12 was sufficient to specifically neutralize 1 μg of either human or murine CXCL12 in leukocyte chemotaxis assays, and has been found *in vivo *to markedly attenuate CXCL12 biology [7,38]. The recombinant human chemokine CXCL12 (PeproTech) and neutralizing anti-CXCR4 antibody (R&D Systems, Minneapolis, MN) were used to assess the proliferation of human RCC cells *in vitro*.

### Human blood specimens

10 mls of heparinized human blood specimens were obtained from each of 21 patients with known mRCC and 2 volunteer normal subjects in a pilot study in accordance with University of California Los Angeles internal review board approval. Buffy coat cells were isolated from each blood specimen and analyzed by FACS analysis for cytokeratin+, CXCR4+, and dual cytokeratin+ CXCR4+ cells.

### Human RCC cell lines

The parental SN12C cell line was originally established in culture from a surgical specimen of human RCC [60]. We chose to use SN12C RCC cell line in our current studies because we have examined the status of the VHL tumor suppressor gene in the parental SN12C cell line and found that intact VHL gene was present in this cell line with no mutations (data not shown). The SN12C parental cells (SN12C-P), SN12C cells stably transfected with vector controls expressing GFP (SN12C-VC), and SN12C-VC cells stably transfected with a lentiviral deliver system to express shRNA to knockdown VHL and continue to express GFP (SN12C-VHL-KD) were generated as previously described [61].

### Heterotopic and orthotopic human RCC-SCID mouse chimeras

Six- to eight-week-old female CB17-SCID beige mice (UCLA Core Facility) were subjected to injection of either 10^4 ^SN12C-VHL-KD or SN12C-VC cells into the subcapsular region of the left kidney (orthotopic model), or 10^6 ^SN12C-VHL-KD or SN12C-VC cells into the flank (heterotopic model). Both the SN12C-VHL-KD and SN12C-VC cells used in this study had been stably transfected with vectors expressing GFP and had been shown to be GFP+ in 100% of cells by both fluorescence microscopy and FACS. For CXCL12 depletion studies, tumor-bearing mice were intraperitoneally injected with either neutralizing anti-CXCL12, preimmune serum, or received no treatment, using a modification as previously described [7,62-64]. Mice bearing tumors were sacrificed after 4 weeks, and their major organs were harvested and processed to assess single cell suspensions for the expression of GFP by FACS analysis.

### FACS analysis

FACS analysis was performed as previously described [7,59] using primary antibodies to CXCR4 (R&D Systems) and pan-cytokeratin; and with secondary antibody Alexa 488 (Molecular Probes, Eugene, OR). For each FACS analysis, 10,000 events per stained condition were analyzed. GFP-expressing cells were analyzed directly without staining.

### RNA isolation and Real-Time PCR

Total RNA was isolated with TRIzol (Life Technologies, Rockville, MD) following manufacturer's instructions as previously described [7,35,59]. Next, 1.5 μg of RNA was used and DNase treated to remove contaminating DNA prior to reverse transcription to cDNA using a ProSTAR First Strand RT-PCR kit per manufacturer's instructions (Stratagene). Subsequently, the cDNA was assessed for changes in CXCR4 expression by real-time PCR using the ABI Prism 7700 sequence detector and SDS analysis software (Applied Biosystems, Foster city, CA) as previously described [7,35,59].

### Hypoxia treatment and whole cell or nuclear extract preparation

Hypoxia treatment of SN12C-P, SN12C-VC and SN12C-VHL-KD cells was performed using a modification as previously described [35]. Briefly, cells were cultured to a density of approximately 80% in complete media and then transferred to starvation media in efforts to synchronize cell cultures. Next, cells were exposed to either normoxia (room air and 5% carbon dioxide) or hypoxia (94% nitrogen, 5% carbon dioxide and 1% oxygen) in Modular Incubator Chambers (Billups-Rothenberg, Inc., Del Mar CA) for the times indicated. Subsequently, whole cell extracts (WCE) or nuclear extracts were prepared as previously described [35]. Briefly, WCE lysis buffer comprised 20 mM HEPES pH 7.9, 25% glycerol, 420 mM NaCl, 1.5 mM MgCl2, and 0.2 mM EDTA, plus a panel of protease and phosphatase inhibitors (PMSF, DTT, and NaF at 1 mM; aprotinin, leupeptin, pepstatin, and β-glcerophophate at10 μg/ml). Buffer for extraction of the nuclear fraction was composed of 20 mM HEPES, pH 7.9, 400 mM NaCl, and 1 mM EDTA.

### Western blotting

Immunoblotting was performed on 40 μg of total protein from either WCE or nuclear extracts as previously described [35]. Briefly, following SDS-PAGE, the proteins were electrophoretically transferred to a PVDF membrane at 100 V for one hour at room temperature and then blocked in BLOTTO for 30 minutes. The membranes were incubated overnight at 4°C with either rabbit anti-human CXCR4 (1:500; Oncogene Research Products, Cambridge, MA) or mouse anti-human HIF-1α antibody (1:500; BD Biosciences). Subsequently, the blots were washed in TTBS and then incubated with either donkey anti-rabbit or goat anti-mouse horseradish peroxidase-conjugated secondary antibodies for 45 minutes at room temperature. After washing in TTBS (3 times, 15 minutes each wash), the immunoreactive proteins were finally visualized using ECL Plus following the manufacturer's instructions (Amersham Biosciences, Piscataway, NJ). To demonstrate equal loading of protein from WCE, the membranes were then stripped and reprobed with a GAPDH antibody (1:500; Abcon).

### Tumor cell chemotaxis

SN12C-P, SN12C-VC, and SN12C-VHL-KD cells exposed to either normoxia or hypoxia for 24 hours were analyzed for chemotaxis in response to CXCL12 as previously described [35]. Briefly, cells were harvested by trypsinization, counted, and resuspended in RPMI 1640 media containing 10% FCS at a concentration of 10^6^/ml. Neuroprobe filter (5 μm diameter) pretreated with 5 μg/ml fibronectin and 12-well chemotaxis chambers were used for these assays. Recombinant CXCL12 (30 ng/ml; Peprotech) was added to the lower wells and 1 × 10^5 ^cells were added to each of the upper wells. The chemotaxis chambers were then incubated for 6 hours at 37°C. After fixing in methanol and staining in 2% Toluidine blue, the number of cells that had migrated through to the underside of the filters was calculated by counting the total number of cells in 5 separate fields of view under 400X magnification.

### Chromatin immunoprecipitation assay (ChIP)

SN12C-VC and SN12C-VHL-KD 2 × 10^6 ^cells were cultured in 10 ml of starvation media in 100 mm dishes for 24 hours before exposure to either normoxia or hypoxia for 4 hours. After the exposure, cells were fixed directly by adding 270 μl of 37% formaldehyde to 10 ml of culture media and incubating at room temperature for 10 minutes. The fixed cells were harvested and prepared for immunoprecipitation using the protocol of ChIP assay kit (Upstate Biotechnology Inc., Lake Placid, New York) with minor modifications. Each sample was precleared with 60 μl of salmon sperm DNA/protein A agarose (Upstate Biotechnology Inc.). 50 μl of the precleared supernatant from each sample was taken for later use in PCR analysis to show total DNA input. One half of each sample was subsequently incubated with either 2 μg of mouse anti-human HIF-1α antibody (BD Biosciences) or no antibody at 4°C overnight. Immune complexes were collected with salmon sperm DNA/protein A-agarose for 1 hour, washed 5 times, and finally eluted in 1% SDS, 0.1 M NaHCO_3_. The eluted immune complexes were subsequently reverse cross-linked and purified by phenol/chloroform extraction. 10 μl of purified DNA from each sample and DNA input were used for PCR analysis. PCR analysis was carried out using primers specific for human CXCR4 promoter sequences. Sequences of promoter-specific primers included the CXCR4 promoter region -1860 to -1578 as follows: sense, 5'-TCGTGCCAAAGCTTGTCCCTG-3'; and anti-sense, 5'-GCGGTAACCAATTCGCGAATAGTGC-3'.

### Transient transfections

SN12C-VC and SN12C-VHL-KD cells were cultured to a density of 80% in complete media in 12-well plates and co-transfected with 0.15 μg of a 2.6-kilobase sequence of the CXCR4 promoter, 0.08 μg of the Renilla control construct (pRL-SV40; Promega, Madison, WI), and 0.08 μg of a HIF-1α construct as previously described [35]. A GFP construct was used to equalize the DNA transfection load. Transfections were performed with Lipofectamine 2000 and Opti-MEM media (Invitrogen, Carlsbad, CA) by following the manufacturer's instructions. The cells were then cultured for 24 hours at 37 °C before exposure to normoxia or hypoxia for 8 hours. After the exposure, cell extracts were made using the luciferase reporter lysis buffer (Promega). Each lysate was subsequently assayed in the dual luciferase reporter assay (Promega) following the manufacturer's instructions; luciferase activity was determined using a Monolight series 2010 luminometer (Analytical Luminescence Laboratory) and then normalized to the Renilla control.

### Proliferation of human RCC cells *in vitro*

To assess the *in vitro *proliferation of human RCC cell lines, SN12C-VC and SN12C-VHL-KD cells were starved overnight in serum-free growth medium, trypsinized, and seeded at a density of 5000 cells/well into 96-well culture plates containing 1% serum and various concentrations (1 ng/ml, 3 ng/ml 10 ng/ml, 30 ng/ml or 100 ng/ml) of CXCL12, or various concentrations (0.5 ug/ml, 2.5 ug/ml or 10 ug/ml) of neutralizing anti-CXCR4 antibody. The cells were allowed to grow for 24, 48 or 72 hours. 1 μCi/well [^3^H]thymidine was added after the incubation, and the cultures were incubated for a further 18 hours. Finally, the cells were harvested using a cell harvester, and [^3^H]thymidine incorporation was quantitated by scintillation counting.

### Quantitation of tumor vessel density

Quantitation of tumor vessel density was performed using a modification as previously described [62]. In brief, tissue sections from tumors of orthotopic human RCC-SCID mice treated with neutralizing anti-CXCL12 or pre-immune serum were dewaxed with xylene and rehydrated through graded concentrations of ethanol. Slides were stained for endothelial cells using polyclonal antibody to Factor VIII-related antigen (Biomeda, Foster City, CA). DAB (Vector Laboratories, Inc., Burlingame, CA) reagent was used for chromogenic localization of Factor VIII-related antigen. After optimal color development, sections were immersed in water and cover-slipped. Tumor specimens were scanned at low magnification (40×) to identify vascular hot spots. Areas of greatest vessel density were then examined under higher magnification (200×) and counted. Any distinct area of positive staining for Factor VIII-related antigen was counted as a single vessel. Results were expressed as the number of vessels per high power field (200×).

### Measurement of *in vivo *apoptosis and proliferation of human RCC

Sections of orthotopically injected SN12C-VC and SN12C-VHL-KD tumors from SCID mice treated with either control or anti-CXCL12 antibodies were stained using either the TdT-mediated dUTP nick end labeling (TUNEL, Roche Applied Science, Indianapolis, IN) method to detect apoptosis or anti-proliferating cell nuclear antigen (PCNA, DAKO Corp, Carpinteria, CA) to detect proliferation, as previously described [62]. Briefly, cells were counted in high power fields per tumor (400×) after scanning at low power (40×) to select areas devoid of frank necrosis. Results were expressed as the number of apoptotic nuclei per 400X field, or the number of proliferating (PCNA positive) cells per 400X field. Similarly, *in vitro *apoptosis of SN12C-VC and SN12C-VHL-KD was studied by plating cells on slide wells with various concentrations of CXCL12 (1 ng/ml, 3 ng/ml 10 ng/ml, 30 ng/ml or 100 ng/ml) or anti-CXCR4 antibody (0.5 ug/ml, 2.5 ug/ml or 10 ug/ml). Cells were stained using the TUNEL assay, and counted in high power fields (400×).

### Statistical analysis

The animal studies involved 6 mice for each treatment group. Comparisons were evaluated by Student's *t *test. Results were presented as means ± SEM. Data were analyzed using the Statview 5.0 statistical package (Abacus Concepts, Berkeley, CA). Data were considered statistically significant if *p *values were < 0.05.

## Competing interests

The author(s) declare that they have no competing interests.

## Authors' contributions

JP carried out the HIF expression studies and helped draft the manuscript. JM carried out the transcription assays and helped draft the manuscript. MDB carried out the human and animal studies. RJP carried out the CXCR4 expression studies. GVT designed and provided the RCC cell lines. KR acquired patient samples and data. JAB provided intellectual input. RMS conceived of the study, and participated in its design and coordination and helped to draft the manuscript. All authors read and approved the final manuscript.
